# Prototype of a scaled‐up microbial fuel cell for copper recovery

**DOI:** 10.1002/jctb.5353

**Published:** 2017-07-24

**Authors:** Pau Rodenas Motos, Gonzalo Molina, Annemiek ter Heijne, Tom Sleutels, Michel Saakes, Cees Buisman

**Affiliations:** ^1^ Wetsus European Centre of Excellence for Sustainable Water Technology Oostergoweg Leeuwarden The Netherlands; ^2^ Sub‐Department of Environmental Technology Wageningen University Wageningen The Netherlands

**Keywords:** MFC, copper, MET, BES

## Abstract

**Background:**

Bioelectrochemical systems (BESs) enable recovery of electrical energy through oxidation of a wide range of substrates at an anode and simultaneous recovery of metals at a cathode. Scale‐up of BESs from the laboratory to pilot scale is a challenging step in the development of the process, and there are only a few successful experiences to build on. This paper presents a prototype BES for the recovery of copper.

**Results:**

The cell design presented here had removable electrodes, similar to those in electroplating baths. The anode and cathode in this design could be replaced independently. The prototype bioelectrochemical cell consisted of an 835 cm^2^ bioanode fed with acetate, and a 700 cm^2^ cathode fed with copper. A current density of 1.2 A/^−2^ was achieved with 48 mW m^−2^ of power production. The contribution of each component (anode, electrolytes, cathode and membrane) was evaluated through the analysis of the internal resistance distribution. This revealed that major losses occurred at the anode, and that the design with removable electrodes results in higher internal resistance compared with other systems. To further assess the practical applicability of BES for copper recovery, an economic evaluation was performed.

**Conclusion:**

Analysis shows that the internal resistance of several lab‐scale BESs is already sufficiently low to make the system economic, while the internal resistance for scaled‐up systems still needs to be improved considerably to become economically applicable.© 2017 The Authors. *Journal of Chemical Technology & Biotechnology* published by John Wiley & Sons Ltd on behalf of Society of Chemical Industry.

## INTRODUCTION

Bioelectrochemical systems (BESs) enable recovery of electrical energy from a wide range of substrates and utilize these electrons to produce electrical power or produce valuable products at the cathode such as hydrogen, acetate or methane.^1^, ^2^, ^3^ Another interesting application is the recovery of metals at the cathode.^4^ BESs for the recovery of metals have several advantages over traditional technologies like electroplating, the most important being the required energy input. The dimensionally stable anodes used in electroplating are designed for the oxidation of water, which requires a high energy input of 2.71 kWh kg^−1^ Cu. By contrast, the anode in BESs uses microorganisms that oxidize organic matter from waste streams to produce an electric current. The overall reaction of acetate oxidation and copper reduction is spontaneous, and therefore electrical energy is generated instead of consumed.^4^, ^5^ When rates are not sufficiently high, an external power supply can be coupled to the cell to increase the current density beyond the short circuit current, so that the copper plating rate is increased further.

The concept of copper recovery using BESs was first demonstrated by Ter Heijne et al., and improvements in cell design have resulted in a current density of 25 A m^−2^ and a power production of 5.5 W m^−1^. In this improved system, copper was recovered at the surface of an electrode with a purity of 99%.^5^ As an alternative for acetate, electron donors present in mining waste streams, like for example tetrathionate,^6^, ^7^, ^8^ have been tested for their suitability as bioanodes. In remote mining locations when waste streams are not available in the vicinity of metal streams, hydrogen, which is produced on site, could be used as an electron donor.^9^ Also, (bio)ethanol could be transported to these locations as a cheap organic alternative to acetate. However, so far, the performance of BESs, using these alternative electron donors, is lower compared with acetate.

While lab scale systems show promising performance, it is necessary that BESs are also tested at larger scale to assess their viability for industrial application.^10^, ^11^ A pertinent challenge for (bio)electrochemical cells is to design scaled‐up systems with the same low internal resistance as labscale devices.^11^, ^12^ For low internal resistance, placement of anode and cathode electrodes at short distances is necessary. For the recovery of metals using BESs, additional criteria are of importance for scaling‐up. For instance, the recovered metals on the cathode need to be removed from the system regularly, while the bioanode has to be kept separated from the metal stream due to metal toxicity for the electro‐active biofilm. The electrochemical baths and removable cathode plates used in the electroplating industry were taken as a model for the design of the prototype reactor. The bioanode was designed as a separate module immersed in between two cathode electrodes in an electrochemical bath. This design enables the anode module as well as the cathode plates to be substituted when required. These removable electrodes, however, do come at a cost of internal resistance, as electrodes cannot be placed as close to each other compared with non‐removable electrodes. The high value of the recovered copper could allow for higher internal resistance compared with other products in BESs with lower value.

The aim of this study was to operate the prototype BES with removable electrodes for copper recovery and to assess its suitability for further scaling‐up. The performance of the prototype was analyzed via polarization curves, and the contribution of each component (anode, electrolytes, membrane and cathode) to the total internal resistance was determined. Finally, an economic assessment was performed to show the potential of BESs for metal recovery using different types of electron donor at the anode.

## MATERIALS AND METHODS

### Design of the prototype BES

**Figure 1:**
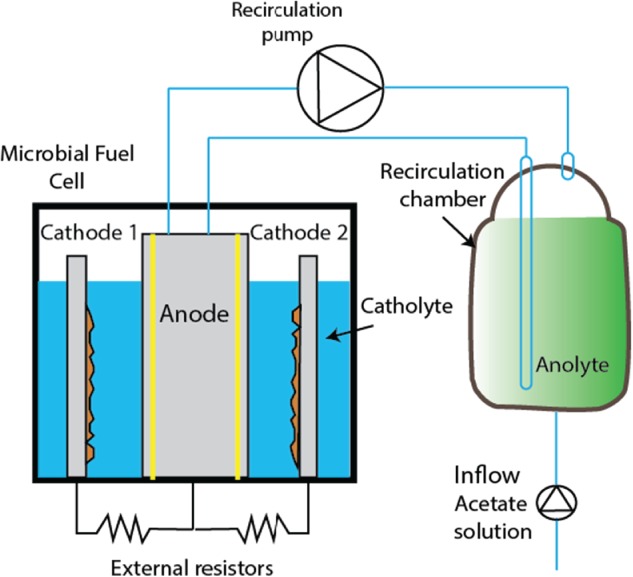
Scheme of the experimental setup (not to scale).

The cathode compartment of the BES represented a traditional plating bath, in which a separate module was placed containing the anode (Fig. 1). The cathode compartment consisted of a PP housing with a volume of 32 L and was placed on stirring plates to allow mixing of the catholyte. Two stainless steel plates served as the cathode electrodes. These cathodes could be lifted to be removed from the cathode chamber. Each cathode had a total (submerged) working surface area of 700 cm^2^ of which only one side (350 cm^2^) was facing the anode module.

**Figure 2:**
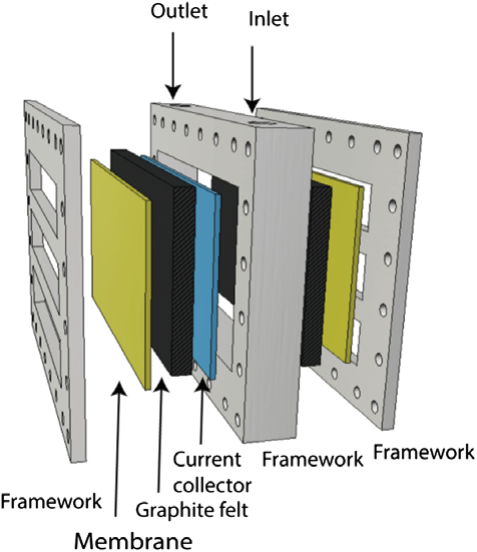
Schematic representation of the bioanode module housing.

The anode compartment was designed as a module (Fig. 2) that was inserted in between the two cathodes, with a distance of 3.5 cm between anode and cathode. This anode module consisted of a PP housing (24.6 × 34 cm), in which four layers of carbon felt (4 mm for each layer, surface area 1–2 m^2^ g^−1^ – Technical Fibre Products Ltd, Kendal, United Kingdom) were placed on both sides of a platinum coated titanium mesh current collector (thickness 1 mm – Magneto Special Anodes BV, Schiedam, The Netherlands). On both sides of this anode an anion exchange membrane (Ralex, MEGA a.s., Czech Republic) was placed, making the anode a separate, removable module. The two membranes combined had a projected surface area of 600 cm^2^. To allow in and outflow of the anolyte, a spacer material (PETEX, Goor, The Netherlands) was placed in between the anode felt and the membrane resulting in an anode compartment volume of 300 mL. The spacer also allowed for a perpendicular flow of the anolyte through the anode. The anolyte was circulated over the anode module and an external circulation bottle (1 L) at a rate of 500 mL min^−1^. The influent was fed to the circulation bottle at a rate of 4 mL min^−1^ giving a theoretical hydraulic retention time (HRT) of 325 min. The anolyte temperature was kept at 30 °C through a heat jacket around the anolyte circulation bottle. The rest of the system was operated at room temperature (23 °C). Figure 1 shows the schematic design of the prototype with the recirculation bottle, pumps and electrical connection.

### Electrolyte composition and inoculums

During start‐up and during the experimental procedure, the anode compartment was fed with acetate solution (20 mmol L^−1^) furthermore containing 0.68 g L^−1^ KH_2_PO_4_, 0.87 g L^−1^ K_2_HPO_4_, 0.74 g L^−1^ KCl, 0.58 g L^−1^ NaCl, 0.28 g L^−1^ NH_4_Cl, 0.1 g L^−1^ MgSO_4_.7H_2_O, 0.1 g L^−1^ CaCl_2_·2H_2_O and 0.1 mL L^−1^ of a trace element mixture.^13^ The system was inoculated with 200 mL of effluent from a running MFC fed with acetate.

During start‐up, the catholyte compartment contained a buffer solution containing 0.68 g L^−1^ KH_2_PO_4_, 0.87 g L^−1^ K_2_HPO_4_ (pH 6.8). Air was continuously supplied to the catholyte compartment to facilitate the oxygen reduction reaction. During this start‐up stage, two external resistors (1000 Ω) were connected in parallel from anode to both cathode electrodes. After 12 days, the cell produced a stable current of 100 mA and was considered ready for the experimental procedure.

After the start‐up phase, three experiments were performed with different copper sulfate concentrations as catholyte (1, 0.5 and 0.1 g L^−1^ Cu^2+^). These copper concentrations in the catholyte correspond to conductivities of 2.2, 2.1 and 1.1 mS cm^−1^, respectively. The anolyte conductivity was 6 ± 0.2 mS cm^−1^ for all experiments. The pH of the catholyte was kept at 4 through the addition of 0.5 mol L^−1^ H_2_SO_4_ every day, while the pH of the anolyte was around 7 ± 0.4. The catholyte was flushed with nitrogen to ensure anaerobic conditions. To this end, floating plastic balls were placed on top of the catholyte to prevent air intrusion.

### Experimental procedure

After the start‐up phase, two external resistors (1000 Ω) were connected in parallel to both cathode electrodes, splitting the cell into two parallel cells sharing one anode. Both resistors were stepwise decreased every 24 h (except in the weekend) until the cell voltage reached a stable value and the resistance was decreased until the cell voltage reached 0 V.

The potentials of the anode and the cathodes were measured using Ag/AgCl reference electrodes (QM711X ProSense QiS, Oosterhout, The Netherlands) placed close to the cathode and in the anolyte outlet. These potentials were continuously recorded using a data logger (RSG40 Memo‐graph, Endress + Hauser), together with the cell voltage (for both cathodes), and the potential of both membranes. Anolyte and catholyte pH were continuously monitored using pH electrodes (Endress + Hauser, CPS41 D). Conductivity was measured externally during sampling of both electrolytes (WTW pH/cond 340i, Weilheim, Germany). Dissolved oxygen was measured using a DO electrode (HQ40d Hach Company, Colorado, USA).

### Analysis

Anolyte, catholyte, and inflow were sampled three times per day to measure organic and inorganic carbon (total carbon analyzer Shimadzu TOC‐VCPH); volatile fatty acids and anion concentrations were measured by Ionic Chromatography (Metrohm 761 Compact IC equipped with a conductivity detector and a Metrosep Organic Acids 6.1005.200 ion exclusion column). Dissolved copper was analyzed by Induction Coupled Plasma (ICP) (ICP‐OES Perkin Elmer Optima 5300). The deposited copper was removed from the electrode by scratching with a knife. The removed copper was then weighed and dissolved in 5 mL of 33% HNO3. This solution was diluted 2000 times and measured by ICP‐OES Perkin Elmer Optima 5300.

### Calculations

The calculation of the anode and cathode Coulombic efficiency and copper recovery were previously described by Ter Heijne et al. and Rodenas et al. 4, 5

The cell voltage generated by the system (E_cell_) is a function of the theoretical voltage (E_emf_) at open circuit conditions, the internal resistance (R_int_) and the current density (J).

(1) $$E_{\text{cell}}=E_{\mathrm{emf}}-JR_{\mathrm{int}}$$

The contribution of each cell component (anode overpotential, cathode overpotential, membrane potential and ionic resistance of both electrolytes) to the internal resistance is described by the equation

(2) $$R_{\mathrm{int}\;}=R_{\text{anolyte}}+R_{\text{Catholyte}}+R_{\text{anode}}+R_{\text{cathode}}+R_{\text{membrane}}$$

The calculation of the contribution of each component has previously been described by Sleutels et al. 14 The total system used in this study consists of two identical circuits (two cathodes) operated in parallel.

### Economic evaluation

We calculated the cost and revenu for different substrates for the recovery of copper in a BES, following the approach in Sleutels et al. 15 to compare with traditional Solvent Extraction/Electro‐Winning (SX/EW). This comparison includes three scenarios where different electron donors are considered: (i) the electron donor is available onsite (wastewater); (ii) bio‐ethanol as electron donor; and (iii) hydrogen which is produced on‐site through steam reforming. For the first scenario, we assume that all the organic matter is hydrolyzed to acetate, being the electron donor to be consumed. Since the electron donor originates form waste, it can be considered free of charge. For the second scenario, bio‐ethanol was used as fuel for its future use in BESs to be used in remote locations as an alternative to other fuels like diesel.^16^ For the third scenario hydrogen was used as the electron donor, as it is produced in many metal industry locations. The use of hydrogen as an interesting alternative electron donor for the recovery of copper in BESs has been previously described by Ntagia et al. 9

Table 1 shows an overview of the parameters used for the calculations and their values The capital cost includes materials for electrodes, current collectors and the membranes. A payback time of 10 years and an interest rate of 6% were considered. The calculations are done for a future optimized system with a specific electrode surface of 100 m^2^ m^−3^. Furthermore, a Coulombic efficiency of 84% for the cathode and 95% for the anode was assumed, obtained in experiments by Ter Heijne et al. 4 For the operational costs of the different electron donor scenarios, the costs for the electron donor and the produced electricity are considered.

**Table 1:** Parameters used for calculations of the economic model of BES for metal recovery

Parameter	Price	Source
Ethanol	0.76 kg^−1^	Alibaba^18^
Copper	4.4 kg^−1^	Alibaba^19^
Hydrogen gas	2.7 kg^−1^	Mueller et al. 20
Electricity	0.06 kWh^−1^	Centraal Bureau voor de Statistiek^21^
BES investment cost	13.6 m^−2^ per year	Sleutels et al. 15
SX/EW	2.29 kg^−1^ Cu	Readette and Marwood^17^

From all the state of the art technologies, SX/EW is the technology that has metallic copper as final product, and it is an electrochemical technique. We use it as a reference industrial application. For the comparison with SX/EW, we took the production cost of 2.29 €/kg Cu described by Readette and Marwood^17^

## RESULTS AND DISCUSSION

### Electrochemical performance of the prototype

**Figure 3:**
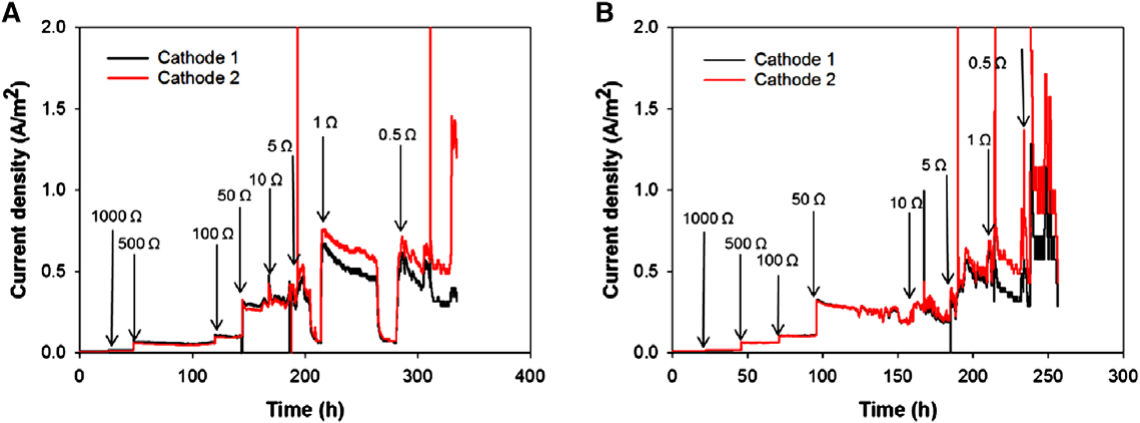
Evolution of current density of duplicate experiments (A and B) in time at different external resistances for cathode 1 (black) and cathode 2 (red) at a copper concentration of 1 g L^−1^. The arrows in these figures indicate when the external resistance was changed.

The performance of the prototype BES was analyzed at three different copper concentrations (1, 0.5 and 0.1 g L^−1^) in the catholyte. In this prototype, the bioanode was connected via two separate wires to the two cathodes, resulting in two parallel currents. Two duplicate experiments were performed with an initial copper concentration of 1 g L^−1^. Figure 3(A) and (B) show the current density as a function of time for these two experiments.

For the first experiment at 1 g L^−1^ (Fig. 3(A)), stable current densities were obtained for both cathodes until the external resistance was reduced to 10 Ω. For cathodes A1 and A2, the current densities dropped drastically at an external resistance of 5 Ω. This drop in current density was related to the limited inflow of acetate due to interrupted inflow of the influent; after feeding acetate, current increased again. The second experiment at 1 g L^−1^ (Fig. 3(B)) showed stable current production at all external resistances.

For the first experiment, the maximum current density achieved by the bioanode was 0.78 A m^−2^, and the current density was higher for cathode 1 (0.78 A m^−2^) than for cathode 2 (0.43 A m^−2^). For the second experiment, the maximum current density was 1.2 A m^−2^, which was higher than the current in the first experiment. In the second experiment, cathode 2 also achieved higher current densities (1.2 A m^−2^) compared with cathode 1 (0.73 A m^−2^).

Also experiments at lower copper concentrations (0.5 and 0.1 g L^−1^) were performed. A reduction in copper concentration led to a decrease in generated current densities; 0.47 A m^−2^ for cathode 1 and 0.48 A m^−2^ for cathode 2 at 0.5 g L^−1^ and to 0.21 A m^−2^ for cathode 1 and 0.27 A m^−2^ for cathode 2 at 0.1 g L^−1^ (data available in supplementary information). The current densities achieved for this prototype are in the same order of magnitude as the current densities obtained on laboratory scale at early stages of the research (∼4 A m^−2^ by Ter Heijne *et al*.). However, the current density for this prototype is one order of magnitude lower than the current densities obtained in a lab cell of 10 cm × 10 cm (∼20 A m^−2^ by Rodenas *et al*.^5^, ^22^

From the data obtained in Fig. 3, also power density curves were constructed (Fig. S1 in the Supporting information) from which the maximum power density was extracted. For the first experiment, the maximum power density was 62 mW m^−2^ for cathode 1 and 53 mW m^−2^ for cathodes 2; while in the second experiment the maximum power density production was 48 mW m^−2^ for both cathodes.

### Comparison of the performance of the prototype with laboratory BES

The performance indicators of maximum current density (*J*_*max*_), maximum power density (*P*_*max*_) and open circuit voltage (*OCV*) for the prototype BES at the three different copper concentrations are summarized in Table 2. For comparison, also the results of an optimized laboratory system are included (5). Both experiments at 1 g L^−1^ had similar open circuit voltages of 0.71 V, however, OCV decreased to 0.68 V at 0.5 g L^−1^ and to 0.61 V at 0.1 g L^−1^ copper in the catholyte. The maximum current density and power density decreased with decreasing copper concentrations. In addition to electrochemical performance, the fate of copper was analysed: while part of the copper was plated on the electrode, another part was lost through chemical precipitation. The remaining share was still in solution at the end of the experiments. The highest copper recovery was 57% for 0.1 g L^−1^ Cu, while 12% of the supplied copper was found as precipitate at the bottom of the reactor. There was no clear relation between copper concentration, copper recovery, and precipitated copper.

**Table 2:** Performance indicators of the prototype with Cu concentrations of 1, 0.5, and 0.1 g L^−1^ in the catholyte compared with the laboratory cell of Rodenas et al.

Experiment	*J*_*max*_	*P*_*max*_	*OCV*	Anode Coulombic efficiency	Copper recovery	Copper precipitated	Internal resistance
	(A m^−2^)	(mW m^−2^)	(V)	(%)	(%)	(%)	(mΩ m^2^)
1 g L^−1^ A	0.78	62	0.707	7	38	29	450
1 g L^−1^ B	1.2	48	0.709	12	32	29	520
0.5 g L^−1^	0.48	52	0.676	6	17	18	942
0.1 g L^−1^	0.27	21	0.610	3	57	12	2110
Rodenas *et al*.	23.0	5500	0.485	26.0	90		10

The maximum anode Coulombic efficiency found in all experiments was only 12%. This low Coulombic efficiciency is most likely due to competing microbial processes for the available acetate, like methanogenesis and sulfate reduction. Acetate was always fed in excess to allow sufficient electron donors for current generation. At the same time, an excess of acetate leads to methane formation.^23^, ^24^ The impact of methanogens can be minimized by limiting the dosing of influent and thereby decreasing the substrate loading to the system.^24^ Furthermore, part of the acetate may have been used for sulphate reduction that is transferred through the anion exchange membrane from the cathode compartment.

### Internal resistance distribution of the prototype

As explained before, current and power densities of the prototype were considerably lower than those achieved in laboratory scale systems.^5^ One of the reasons is the new cell design used here, which allowed the anode module and the cathodes to be removed separately at any moment, without disassembling the complete system, resulting in larger distances between the anode and cathode electrodes than in optimized lab‐scale systems and thus resulting in a larger internal resistance. The total internal resistance of the cell at different catholyte concentrations is shown in Table 2. When the internal resistance for each side of the prototype was calculated from the point at maximum power density, we found that for the first experiment this internal resistance for cathode 1 was 1473 mΩ m^2^ while the internal resistance for cathode 2 was 890 mΩ m^2^. Because of the parallel connection, the resistance of the total prototype was 550 mΩ m^2^ . For the second experiment at 1 g L^−1^ the numbers are similar, cathode 1 had an internal resistance of 931 mΩ m^2^, while the internal resistance of cathode 2 was 678 mΩ m^2^. The total internal resistance of the prototype in experiment 2 was 400 mΩ m^2^. The internal resistance increased at lower concentrations of copper, from 450 mΩ m^2^ at 1 g L^−1^ to 2100 mΩ m^2^ at 0.1 g L^−1^. These resistances are one to two orders of magnitude higher than the internal resistance of 21 mΩ m^2^ reported by Rodenas *et al*.

**Figure 4:**
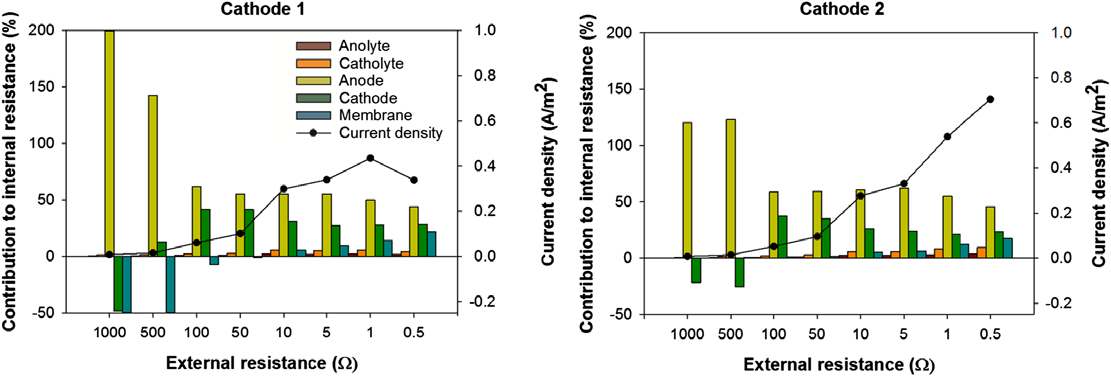
Contribution of partial internal resistance to the total internal resistance; anode (light green bar), cathode (dark green bar), membrane (blue bar), catholyte (orange bar) and anoloyte (red bar) at different external resistances at different external resistances. In addition, the current density at each external resistance is shown.

To have a more detailed analysis of the performance of the system, the contribution of each part of the system to the internal resistance was calculated (Equation (5)). The partial internal resistances were calculated from the overpotentials in the electrodes, and the voltage losses in the membrane and electrolytes at each external resistance.^14^ Figure 4 shows the distribution of the internal resistance for both cathode compartments at different external resistances in the experiment at 1 g L^−1^. This figure shows that the anode had the main contribution to total internal resistance, between 45% and 55% (734 mΩ m^2^ to 850 mΩ m^2^). The second largest contribution to the total internal resistance was by the cathode, between 20% and 35% (209 mΩ m^2^ to 420 mΩ m^2^). Both the ionic and membrane contributions to the total internal resistance were small, although the membrane resistance increased at higher current densities due to the higher rate of ion transport.^25^ It reached a value of 185 mΩ m^2^ for the membrane on the side of cathode 1 and 220 mΩ m^2^ for the membrane on the side of cathode 2.

At high external resistance and thus at low current densities, the voltage contribution of the cathode and in some cases the membrane, showed a negative contribution to the total internal resistance. This might likely be attributed to the presence of dissolved oxygen, since the reduction of oxygen occurs at a higher cathode potential than the reduction potential of copper. Since the standard potential of copper reduction is used in the calculations this leads to negative values for the internal resistance. Although the cathode is sparged with nitrogen gas, the presence of oxygen can be explained by the fact that the cathode compartment is only closed from the air by floating plastic balls on top of the catholyte, and still quite some surface is exposed to air. At lower external resistances and thus at higher current densities, all available oxygen is reduced and on top of that also copper is reduced leading to positive values. In some cases, the membrane potential also shows negative values which are caused by the difference in electrolyte composition in the anode and cathode.

### Comparison of laboratory BES with prototype for upscaling

As shown in the previous part, the prototype BES did not perform as well as the laboratory BES. Table 3 shows the difference between the prototype and the previous laboratory cell design described in Rodenas et al. There are no major differences between the materials, membranes, recirculation speed, HRT or volumes. However, the prototype was designed with four layers of felt on each side of the current collector. By contrast, the cell in Rodenas et al. was designed with only one layer of felt, which had a forced flow of anolyte in a perpendicular direction through the electrode. This forced flow allowed for better mixing and therefore better mass transport in the anode electrode. As mentioned before, the bioanode was the main contributor to internal resistance and the mixing, especially in four layers of felt, is one of the issues that could lead to these losses. When the mixing is not sufficient, not all parts of the electrode have access to substrate and therefore microorganisms will not colonize the full surface of the electrode. Furthermore, the produced protons will not be transported away from the biofilm, leading to higher potential losses. Another issue for this prototype design could have been insufficient current collection. The cassette design only allowed for a small pressure on the felt layers towards the current collector while in the laboratory design the felt was firmly screwed together with the current collector. Finally, there was a difference in the distance between electrodes. In the laboratory cell, the distance between electrodes was seven times smaller than with the prototype, which had 3.5 cm between cathode and anode. Still, even in the prototype design, the contribution of the ionic resistance of the electrolyte was always below 10% of the total resistance.

**Table 3:** Summary of design parameters of prototype compared with Rodenas et al.

Design parameters	Unit	Prototype	Rodenas et al.
Anode material		4 layers graphite felt	1 layer graphite felt
Current collector		Pt coated Ti mesh	Pt/Ir 80:20 wires
Cathode material		Stainless Steel	Copper
Electrode spacing	cm	3.5	0.5
Membrane		AEM	AEM
Surface anode	cm^2^	835	100
Surface cathode	cm^2^	700	100
Volume anolyte	L	1	0.5
Volume catholyte	L	32.2	10
Anolyte recirculation	mL min^−1^	500	200
Catholyte recirculation	mL min^−1^	‐	200
Anolyte HRT	min	325	200
Volume of inoculum	mL	200	200

### Economic evaluation of BESs for metal recovery

In addition to the performance indicators used in Table 2, the economic feasibility of copper recovery in BESs needs to be considered. For BESs to become applicable in industry for the recovery of metals, an evaluation between capital expenditure and cost is required.^26^, ^27^, ^28^ In general, the main disadvantage of BESs compared with SX/EW is the high investment for materials: especially, the electrodes, current collectors, and membranes have a big impact on this investment. Therefore, to be competitive with conventional technologies for metal recovery, BESs need to operate at a high current density to reduce its footprint and, therefore, its specific investment costs. The internal resistance is the dominant factor in determining the current produced by the system.

To see if BESs are competitive with conventional technologies like SX/EW, three different scenarios with different electron donors: (i) wastewater; (ii) bio‐ethanol; and (iii) hydrogen, for the recovery of copper were compared with SX/EW. When these electron donors are used in an MFC, electrical power is produced and this power can either be discharged to the electrical grid (revenue) or used to power the system itself. For the MFC, an open cell voltage of 0.5 V was used for a cell operated on 10 mmol L^−1^ acetate and 1 g L^−1^ copper. The internal resistance in combination with voltage output will define the current density, and this, in turn, determines the amount of required electron donor and recovered copper.

**Figure 5:**
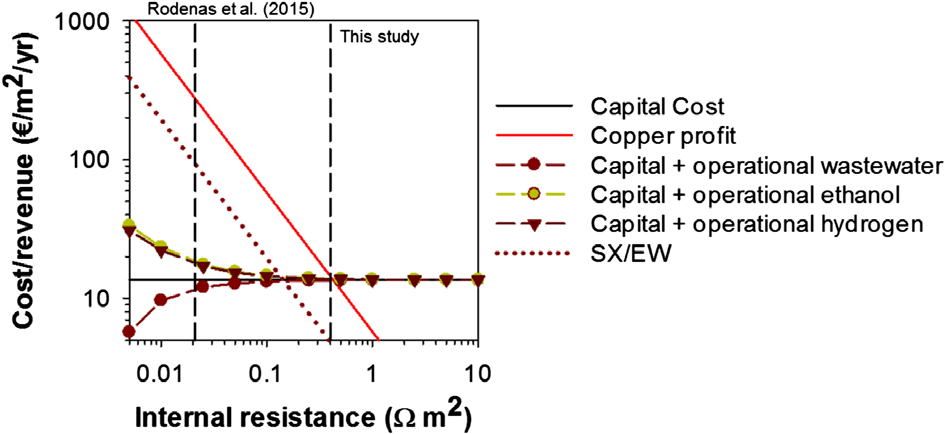
Cost and revenue as a function of the internal resistance for three different electron donors for an MFC with an output voltage of 0.5 V. As a reference technology the costs for SX/EW are included. Also, the results achieved by Rodenas et al. and in this study are shown.

Figure 5 shows the results for the three different business cases in an MFC. This figure shows that the profit for recovered copper is directly related to the internal resistance because the produced current, and thus the produced copper, changes as a fucntion of the internal resistance. For the system itself a capital cost of 13.6 €/m^2^ per year is taken into account. Figure 5 shows the results for the three different scenarios for the electron donors. When wastewater is used as electron donor, which can be considered free of charge, the cost go down at low internal resistance due to the produced electricity. When hydrogen or ethanol are used as electron donor, the costs increase at low internal resistance, since the amount of consumed electron donor, and thus the costs for electron donor, increases even though electricity is produced. For all scenarios, the revenues become higher than the cost when the internal resistance decreases below 400 mΩ m^2^. The most profitable business case is when wastewater is used as the electron donor since this electron donor does not require any additional costs. Actually, this business case would have been even more profitable if the savings on wastewater treatment had been considered. The scenario for both hydrogen and ethanol have similar revenues, since the costs of both electron donors are comparable.

Next to the results for the different electron donors, Fig. 5 also shows the comparison with SX/EW as a competing technology. For SX/EW a constant price per kg of copper was assumed, resulting in a constant negative slope in relation to the increasing internal resistance. The MFC for copper removal becomes attractive as an alternative for SX/EW for an internal resistance lower than 150 mΩ m^2^ when either wastewater or hydrogen are used as an electron donor.

Finally, Fig. 5 shows the internal resistance achieved in the laboratory study and the internal resistance achieved in this study for the prototype. The internal resistance of the laboratory device (21 mΩ m^2^) is one order of magnitude lower than the minimum internal resistance required to break even compared with the copper revenues (400 mΩ m^2^), and also one order of magnitude lower than the resistance of 150 mΩ m^2^ required to be competitive with SX/EW. The minimum internal resistance for the prototype was 450 mΩ m^2^ which is both higher than the required internal resistance to become economically interesting and to be competitive with SX/EW. The challenge will be to show the same performance at industrial scale compared with laboratory scale.

## FUTURE PERSPECTIVES IN CELL CONFIGURATION AND UP‐SCALING

We demonstrated the design and operation of a prototype BESs for the recovery of copper with the potential for upscaling. The design of this prototype was based on the electroplating baths found in electrowinning industry and allowed for flexible replacement of both the anode and the cathode electrodes. This prototype system produced current densities of 1.2 A m^−2^. For the prototype described here we identified the anodic resistance as the major contribution to the internal resistance. The high internal resistance and specifically the high anodic resistance may be caused by: (i) insufficient electrolyte mixing which led to an insufficient use of the available surface area; (ii) non‐optimal current collection from the anode material; and (iii) too high a distance between electrodes leading to a high electrolyte resistance. To come to a suitable future design for the upscaling of BESs, these limitations need to be taken into consideration. Although copper was successfully plated at the cathode and recovered, the bioanode did not work according to expectations compared with high current densities achieved in previous cell designs. The high internal resistance and low Coulombic efficiency may be related to insufficient mass transfer, competing reactions such as methanogenesis, sulphate reduction, or acidification of the biofilm by the formed protons. Research on prototypes of BESs provides important information on the issues related to upscaling of these devices. Solving these critical points could bring BESs closer to practical implication and economic viability.

## Supporting information


**Appendix S1.**

## References

[1] Hamelers HVM , Ter Heijne A , Sleutels THJ , Jeremiasse AW , Strik DPBTB and Buisman CJN , New applications and performance of bioelectrochemical systems. Appl Microbiol Biotechnol 85:1673–1685 (2009). 20024546 10.1007/s00253-009-2357-1

[2] Logan BE , Call D , Cheng S , Hamelers HVMM , Sleutels THJAJA , Jeremiasse AW *et al*, Microbial electrolysis cells for high yield hydrogen gas production from organic matter. Environ Sci Technol 42:8630–8640 (2008). 19192774 10.1021/es801553z

[3] Rabaey K and Rozendal RA , Microbial electrosynthesis ‐ revisiting the electrical route for microbial production. Nat Rev Microbiol 8:706–716 (2015). 10.1038/nrmicro2422 20844557

[4] Heijne A Ter , Liu F , Weijden R van der, Weijma J , Buisman CJN , Hamelers HVM *et al*, Copper recovery combined with electricity production in a microbial fuel cell. Environ Sci Technol Am Chem Soc 44:4376–4381 (2010). 10.1021/es100526g 20462261

[5] Rodenas Motos P , Ter Heijne A , van der Weijden R , Saakes M , Buisman CJN and Sleutels THJA , High rate copper and energy recovery in microbial fuel cells. Front Microbiol. 6:1–8 (2015). 26150802 10.3389/fmicb.2015.00527 PMC4473641

[6] Sulonen MLK , Kokko ME , Lakaniemi AM and Puhakka JA , Electricity generation from tetrathionate in microbial fuel cells by acidophiles. J Hazard Mater 284:182–189 (2008). 10.1016/j.jhazmat.2014.10.045 25463232

[7] Sulonen MLK, Lakaniemi A‐M , Kokko ME and Puhakka JA , The effect of anode potential on bioelectrochemical and electrochemical tetrathionate degradation. Bioresource Technol 226:173–180 (2017). 10.1016/j.biortech.2016.12.023 27997871

[8] Dopson M , Ni G and Sleutels THJA , Possibilities for extremophilic microorganisms in microbial electrochemical systems. FEMS Microbiol Rev 40:164–181 (2016). 26474966 10.1093/femsre/fuv044 PMC4802824

[9] Ntagia E , Rodenas P , Ter Heijne A , Buisman CJN and Sleutels THJA , Hydrogen as electron donor for copper removal in bioelectrochemical systems. Int J Hydrogen Energy 41:5758–5764 (2016).

[10] Logan BE , Scaling up microbial fuel cells and other bioelectrochemical systems. Appl Microbiol Biotechnol 85:1665–1671 (2010). 20013119 10.1007/s00253-009-2378-9

[11] Brown RK , Harnisch F , Wirth S , Wahlandt H , Dockhorn T , Dichtl N *et al*, Evaluating the effects of scaling up on the performance of bioelectrochemical systems using a technical scale microbial electrolysis cell. Bioresource Technol 163:206–213 (2014). 10.1016/j.biortech.2014.04.044 24813389

[12] Premier GC , Kim JR , Michie I , Popov A, Boghani H , Fradler K *et al*, Issues of scale in microbial fuel cells and bioelectrochemical systems. World Renew Energy Forum, WREF 2012, Incl World Renew Energy Congr XII Color Renew Energy Soc Annu Conf. 6:4918–4925 (2012).

[13] Zehnder AJB , Huser BA , Brock TD and Wuhrmann K , Characterization of an acetate‐decarboxylating, non‐hydrogen‐oxidizing methane bacterium. Arch Microbiol 124:1–11 (1980). 6769415 10.1007/BF00407022

[14] Sleutels THJA , Hamelers HVM , Rozendal RA and Buisman CJN , Ion transport resistance in microbial electrolysis cells with anion and cation exchange membranes. Int J Hydrogen Energy 34(9):3612–20 (2009).

[15] Sleutels THJA , Ter Heijne A , Buisman CJN and Hamelers HVM . Bioelectrochemical systems: An outlook for practical applications. ChemSusChem 5:1012–1019 (2012). 22674691 10.1002/cssc.201100732

[16] Kim JR , Jung SH , Regan JM and Logan BE , Electricity generation and microbial community analysis of alcohol powered microbial fuel cells. Bioresource Technol 98:2568–2577 (2007). 10.1016/j.biortech.2006.09.036 17097875

[17] Readette D and Marwood B , 4th Annual Nickel‐Cobalt‐Copper Event ALTA 2013 . Tiger Resources' Kipoi Copper Project Stage Ii ‐ Phased Development of a 50,000Tpa Integrated Agitated Leach, Heap Leach, Sx‐Ew. Perth, Australia (2014).

[18] Dalian BaoDalian Baotai Chemical Co., Ltd . tai Chemical Co. L. Ethanol(Ethyl Alcohol) CAS No.: 64‐17‐5. [Online]. Available: https://www.alibaba.com/product-detail/Ethanol-Ethyl‐Alcohol‐_60402128125.html [16 December 2016].

[19] InfoMine . Copper price (2015). [Online]. Available: http://www.infomine.com/ChartsAndData/ChartBuilder.aspx?z=f&gf=110563.USD.lb&dr=5y&cd=1 [21 November 2016].

[20] Mueller‐Langer F , Tzimas E , Kaltschmitt M and Peteves S , Techno‐economic assessment of hydrogen production processes for the hydrogen economy for the short and medium term. Int J Hydrogen Energy 32:3797–3810 (2007).

[21] CBS . Aardgas en elektriciteit, gemiddelde prijzen van eindverbruikers [Internet]. Statline.Cbs.Nl. (2015). [Online]. Available: http://statline.cbs.nl/StatWeb/publication/?VW=T&DM=SLNL&PA=81309NED&D1=0-1,3,5,7‐8,11,15&D2=0&D3=a&D4=a&HD=121114‐1344&HDR=G1,G2,T&STB=G3 [7 December 2016].

[22] Wang Z , Lim B‐S , Lu H , Fan J and Choi C‐S . Cathodic reduction of Cu2+. Bull Korean Chem Soc 31:2025–2030 (2007).

[23] Sleutels THJA , Darus L , Hamelers HVM and Buisman CJN . Effect of operational parameters on Coulombic efficiency in bioelectrochemical systems. Bioresource Technol 102:11172–11176 (2011). 10.1016/j.biortech.2011.09.078 22004593

[24] Sleutels T , Molenaar S , Heijne A and Buisman C , Low substrate loading limits methanogenesis and leads to high coulombic efficiency in bioelectrochemical systems. Microorganisms Multidisciplinary Digital Publishing Institute (2016). 10.3390/microorganisms4010007 PMC5029512 27681899

[25] Sleutels THJA , ter Heijne A , Kuntke P , Buisman CJN and Hamelers HVM . Membrane selectivity determines energetic losses for ion transport in bioelectrochemical systems. ChemistrySelect 2:3462–3470 (2017).

[26] Fleming CA , Metallurgical S , Sgs C . Platsol Tm Process Provides a Viable Alternative To Smelting. SGS mineral Services (2002).

[27] James PI and Baker M , Profitable copper production from low‐grade waste ores (2015). www.miningengineeringmagazine.com

[28] Di Lorenzo M , Use of microbial fuel cells in sensors In Microbial Electrochemical and Fuel Cells: Fundamentals and Applications *,* 341–356 (2015). [Online]. Available: http://www.sciencedirect.com/science/article/pii/B9781782423751000113 [25 August 2016].

