# Supplementary material for: Prototype of a scaled‐up microbial fuel cell for copper recovery
Source: J Chem Technol Biotechnol. 2017 Jul 24;92(11):2817–24. doi: 10.1002/jctb.5353 (PMC5655933; doi:10.1002/jctb.5353)
Supplement: Supplementary file 1 — Appendix S1. [file JCTB-92-2817-s001.docx]

Prototype of a scaled-up Bioelectrochemical System for copper recovery

### P. Rodenas^1,2^,^,^ Gonzalo Molina^1^; A ter Heijne^2,^*; T.H.J.A. Sleutels^1^, M Saakes^1^, C.J.N. Buisman^1,2^

### 1 Wetsus, European centre of excellence for Sustainable Water Technology, Oostergoweg 9, 8900 CC Leeuwarden, The Netherlands

### 2 Sub-Department of Environmental Technology, Wageningen University, Bornse Weilanden 9, 6708 WG Wageningen, The Netherlands

#### ^*^ corresponding author: Annemiek.terheijne@wur.nl

Supplementary information


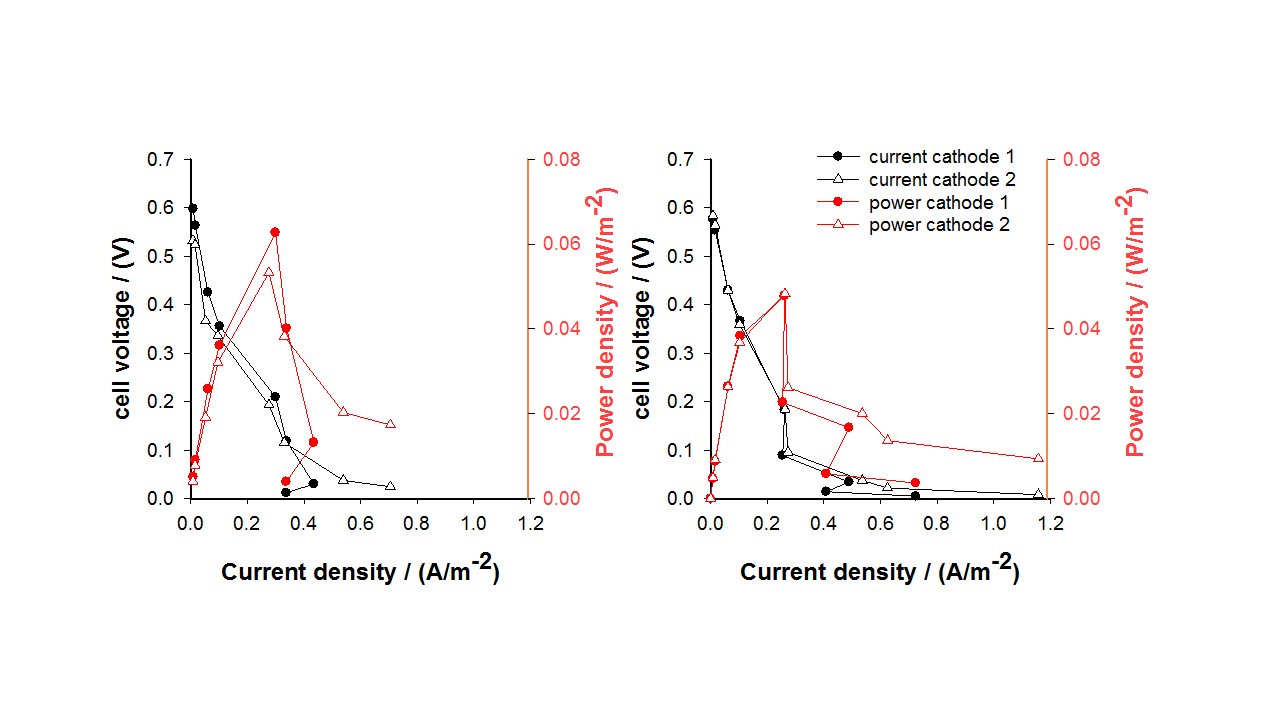


Figure S1 Polarization and power curves of the two different sides of the biocassette known as cathode 1 and cathode 2 at an initial copper concentration of 1 g L^-1^.

Figure S2. Polarization (black) and power (red) curves of the two different sides of the biocassette known as cathode 1 (black dot) and cathode 2 (white dot) at an initial copper concentration of 0.5 g/L

Figure S3. Polarization (black) and power (red) curves of the two different sides of the biocassette known as cathode 1 (black dot) and cathode 2 (white dot) at an initial copper concentration of 0.1 g/L

## Calculations business case scenarios

This model used the following assumptions:

Cathodic coulombic efficiency (η_cathode_) of 95%

Anodic coulombic efficiency (η_anode_) of 84%

We assume an investment of capital cost (Inv) of 13.6 €/m^2^ per year

This model used the parameters described in table 1 to calculate the price (Pr, €/Kg)).

Calculation of the current density:

To calculate the current density, we assume an equivalent circuit of a voltage source with an internal resistance in series.

$J(A/m^{2})=-\frac{V_{cell}(V)-V_{OC}(V)}{R_{\mathrm{int}}(\Omega\cdot m^{2})}$ (A.1)

Where J is the current density measured in A/m^2^, V_OC_ is the voltage at open circuit of the cell measured in volts (V), Vcell is the voltage output or voltage applied to the cell measured in volts (V) and Rint is the internal resistance measured in Ω.m^2^.

Calculation of the amount of amount of electrons per year:

$X(mole^{-}/year)=\frac{J(A/m^{2})\cdot t(s/year)}{F(C/mol)}$ (A.2)

Where X is the number of electrons moved through the equivalent circuit in a year. t is the number of seconds in a year, and F is the Faraday constant (96485 C/mol).

Calulation of the electricity revenue (Ele)

$Ele=\frac{X(mole^{-}/year)\cdot(-V_{cell}(V))\cdot F(C/mol)\cdot0.06(€/kWh)}{3600(s/hour)\cdot1000(W/kW)}$ (A.3)

Calculation of the cost of an electron donor:

$\text{Cost}_{edonor}(€/year\cdot m^{2})=\frac{X(mole^{-}/year)\cdot\nu\cdot\Pr(€/Kg)}{Mm(mol/Kg)\cdot\eta_{anode}}+Inv+Ele$ (A4)

where Cost_edonor_ is the cost of the electron donor in a year per m^2^ of elèctrode area, ν is the stoichiometry coefficient for the oxidation reaction related to the number of electron mols per mol of electron donor, Pr is the price of the electron donor defined in Table 1, Inv is the investment required for the capital costs of the BES reactor, Mn is the molar mass of the electron donor molecule and ƞ_anode_ is the coulombic efficiency of the electron donor oxidation.

Calculation of the amount of Kg of copper per year: The mass of metal deposited in a year of electroplating can be calculated using equation A.5

$\text{Mass}_{eaccep}(Kg/year\cdot m^{2})=\frac{X(mole^{-}/year)\cdot\nu}{Mm(mol/Kg)\cdot\eta_{cathode}}$ (A.5)

Were Mass_eaccep_ is the amount of metal deposited over the a square meter electrode in a year (kg/year·m^2^)

Calculation of the copper profit:

$\text{Rev}_{eaccep}(€/year\cdot m^{2})=\frac{X(mole^{-}/year)\cdot\nu\cdot\Pr(€/Kg)}{Mm(mol/Kg)\cdot\eta_{cathode}}$ (A.6)

where Rev_eaccep_ is the revenue of the electron acceptor in a year per m^2^ of elèctrode area, ν is the stoichiometry coefficient for the oxidation reaction related to the number of electron mols per mol of electron donor, Pr is the price of the electron acceptor (copper) defined in Table 6.2, Mn is the molar mass of the electron acceptor and ƞ_cathode_ is the coulombic efficiency of the electron donor oxidation.

To compare with the state of the art technology SX/EW we calculate the cost of the copper using this technology by equation A7

 (A.7)

Where the SX/EWcost is the cost of SX/EW technology is calculated from the production cost and the mass of metal produced in a year per area of electrode.
